# A case report of semitendinosus tendon autograft for reconstruction of the meniscal wall supporting a collagen implant

**DOI:** 10.1186/2052-1847-5-4

**Published:** 2013-03-28

**Authors:** Juan D Ayala Mejias, Roselyn C Alvarez Sciamanna, Manuel Perez-España Muniesa, Luis Alcocer Pérez-España

**Affiliations:** 1 Department of Orthopaedic Surgery, Hospital San Rafael, Madrid, Spain; 2 Department of Orthopaedic Surgery, Hospital Asepeyo, Madrid, Spain; 3 Department of Orthopaedic Surgery, Hospital Infanta Leonor, Madrid, Spain; 4 Sanatorio Quirúrgico Virgen del Mar, Madrid, Spain

**Keywords:** Meniscectomy, Meniscal wall, Autograft, Semitendinosus tendon, Collagen implant

## Abstract

**Purpose:**

Describe the evolution of the reconstruction of meniscal rim with semitendinosus tendon in a patient with knee pain after a subtotal meniscectomy and absence of meniscal wall.

**Method:**

32 years old male with a six-month history of the left knee pain after a subtotal meniscectomy. The MRI indicated a small internal meniscal remainder without posterior horn attachment. Taking this absence as a relative contraindication for implant and meniscal transplantation, the reconstruction of a new meniscal wall with semitendinosus tendon autograft was considered. A collagen meniscal implant was attached to the new wall five months later.

**Results:**

After two years the patient referred only non specific discomfort with full pain relief in the medial compartment. The MRI revealed integration of implants without significant degenerative changes compared to previous images.

**Conclusions:**

This staged technique was designed to restore medial meniscus-like biologic tissue in a symptomatic patient following arthroscopic subtotal meniscectomy with a significant loss of the peripheral meniscus rim. Symptomatic improvement was obtained at two years follow-up.

## Background

The meniscus is an important structure for the normal function of the knee*. A partial or total meniscectomy develops or accelerates the joint’s degenerative changes*[1,2]. This structure transmits and distributes part of the load and increases the contact area between femoral condyle and tibial plateau. A meniscectomy decreases the contact area and increases the local peak load leading to cartilage damage and further joint degeneration [3]. *In the mid- and long-term meniscal repair has been shown to have success rates of 70%-80%. Despite these excellent results, recent data reported that meniscal repair is only considered in 3%-5% of meniscal surgeries*[4]*.*

*When meniscal repair is not indicated* several therapeutic options have been reported for the meniscal replacement in order to prevent osteoarthritis, including meniscal collagen implant or CMI, with good clinical and radiological results in short and long term follow-up studies [5-8]. However, this procedure requires meniscal wall tissue to stimulate cells and tissue migration [9]. Different materials and techniques have been developed, not always with satisfactory results [10-13].

Here is presented the complete description of a case, the surgical technique and the results after two years of follow-up of a symptomatic patient after a subtotal meniscectomy of the medial meniscus with a complete loss of the meniscal wall. Taking this absence as a relative contraindication for implant and meniscal transplantation [14], the reconstruction of a new meniscal wall with semitendinosus tendon autograft was considered as a surgical alternative.

## Case presentation

### Patient’s background

A 32 year old man was referred because of left knee pain. Six months before he had a subtotal meniscectomy of the medial meniscus for a bucket handle tear. *He was a local policeman that could not develop his work because of non-responsive pain in prolonged stand up position and severe limitation for running and other sports activities.*

Physical examination revealed a varus morphotype with positive meniscal maneuvers and medial joint pain. Radiographic imaging showed impingement of the medial compartment corresponding to a Fairbank grade II. The report of magnetic resonance imaging indicated a small medial meniscus remnant, almost absent in body and no posterior horn attachment with a focal osteochondritis of the medial femoral condyle. An arthroscopic evaluation was recommended and different therapeutic options were explained.

### Surgical technique

**Figure 1:**
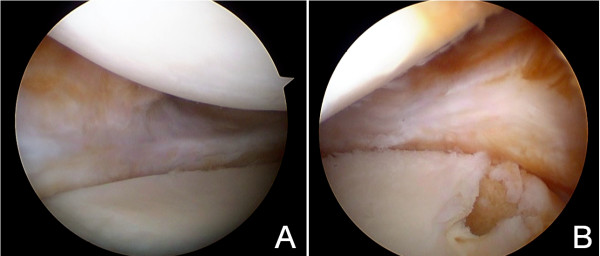
**Post-meniscectomy arthroscopy. A**. Middle area of medial compartment 6 months after a total medial meniscectomy. No meniscal rim is observed, only a small reparative tissue is seen and had to be removed. **B**. Posterior area of medial compartment. Tunnelization of posterior tibial tunnel is performed.

**Figure 2:**
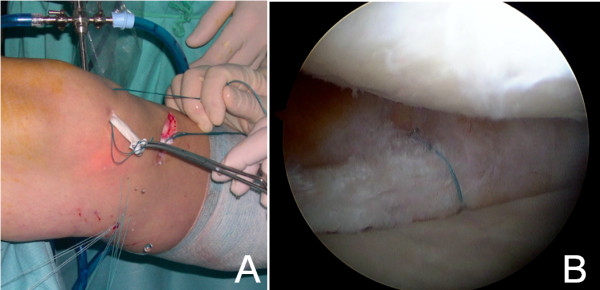
**Semitendinosus graft technique. A**. Semitendinosus autograft is inserted through the antero-medial portal and fixed with several sutures “inside-out” and “outside-in”. Observe the pulling sutures through the tibial tunnel. **B**. Arthroscopic view of semitendinosus tendon once passed the first suture.

After informed consent and pre-surgical study, knee arthroscopy was performed under epidural anesthesia and preventive ischemia of the left lower extremity. Arthroscopy showed absence of part of the body and posterior horn of medial meniscus (Figure 1A and 1B). The anterior part of the meniscal tissue showed degenerative signs. In the lateral area of the medial femoral condyle near the notch, a focal Outerbridge chondral injury grade III was observed. The remaining meniscal tissue was regularized until reaching a healthy-look tissue and the meniscal wall was reconstructed with autologous graft with a double semitendinosus tendon. The tendon was identified through an oblique incision over the tibial insertion of the hamstrings. The graft was introduced through the antero-medial portal across the anterior capsule, pulling on the sutures through a tibial tunnel that was previously drilled in antero-posterior and medio-lateral direction pointing to the insertion of the posterior horn (Figures 1B and 2A). The graft was fixed with sutures “outside-in” to the menisco-tibial ligaments and then sutured to the anterior horn remainder, “inside-out” in the body, “all inside” in the posterior horn and a tibial staple distally to the tibial tunnel (Figure 2A and 2B).

**Figure 3:**
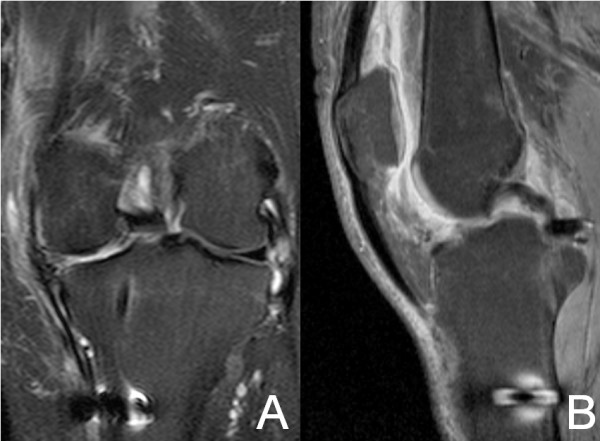
**MRI in T1 sequences after semitendinosus graft implantation.** In coronal plane (3**A**) the double semitendinosus tendon reaches the lateral margin of the lateral tibial plateau where CMI will be attached. In sagittal plane (3**B**) the posterior graft is adapted to the posterior tibial plateau near the PCL insertion.

The knee was immobilized at 20 degrees of flexion for 3 weeks. *Partial weight bearing was authorized at the third post-operative week.* Then, range of motion was allowed from 0 to 90 degrees until the sixth week. Postoperative physical examination was made, showing only mild effusion of the knee. After six weeks, progressive flexion was permitted. *A 1.5 Tesla MRI in coronal plane (Figure*3*A) showed that the double semitendinosus tendon reached the lateral margin of the lateral tibial plateau. In sagittal plane (Figure*3*B) a bulky posterior graft was adapted to the posterior tibial plateau.*

**Figure 4:**
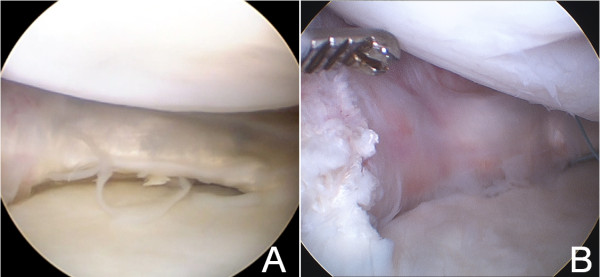
**Arthroscopic view before CMI implantation.** Arthroscopic view of posterior (4**A**) and anterior (4**B**) zones of semitendinosus tendon covered by sinovial tissue five months after the implantation.

**Figure 5:**
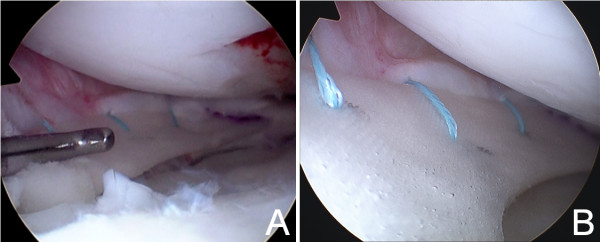
**Arthroscopic view after CMI implantation.** CMI inserted in posterior (5**A**) and anterior (5**B**) zones of the new “meniscal wall”.

Five months later he underwent a new arthroscopy. The new reconstructed “meniscal wall” with semitendinosus tendon was stable and invaded by a synovial tissue similar to the native tissue (Figure 4A and 4B). The anterior horn showed an oblique tear with degenerative appearance in the graft union and the osteochondral lesion reduced its size with some fibrocartilage areas. The anterior horn of medial meniscus was regularized and a collagen meniscal implant of 40 mm was inserted. The CMI was attached to the new *meniscal rim* with sutures “all-inside” in the posterior horn (Figure 5A) and sutures “outside-in” for the body and anterior horn (Figure 5B). In addition, microfractures were performed in the area of osteochondritis with stabilization of the edges of the cartilage defect with a radiofrequency terminal.

### Postoperative follow up

In the postoperative period, the knee movement was limited from 0° and 30° during the first week, until 60° between first and third week and up to 90° between third and sixth week. Then, free range of motion was allowed and a progressive partial weight bearing was authorized in the third post-operative week.

Two months after the last surgery, the patient’s physical examination revealed a joint range of motion of 0º to 120°, without effusion, moderate quadriceps atrophy and some occasional discomfort related with sustained load bearing.

After 3 months, the patient was admitted in other Hospital because of an acute truncal viral encephalitis of unknown etiology. However, after a complete neurological examination any correlation with the knee surgery was discarded.

At 5 months, the patient only presented occasional discomfort in the popliteal region, mild atrophy of the quadriceps and minimum non inflammatory effusion that was drained and sent to microbiological study. The magnetic resonance imaging demonstrated that both grafts were correctly placed in middle and posterior zones with moderate anterior extrusion and marked synovitis. In the sample cultures, there was growth of colonies of S*taphylococcus simulans. Despite that the culture results were considered to be a contaminating agent rather than a pathogen, antibiotic therapy was prescribed according to the antibiogram.*

At 7 months, a diagnostic arthroscopy was performed because of persistent knee effusion with no local or systemic signs of infection. A marked generalized synovitis was observed, and three samples were taken for pathologic and microbiologic studies. Then, a partial synovectomy and joint toillette was performed. The meniscal implant was stable with complete integrity. The chondral defect of medial femoral condyle was covered with fibrocartilage. The results of the studies reported inflammatory synovitis with no pathogens growth.

**Figure 6:**
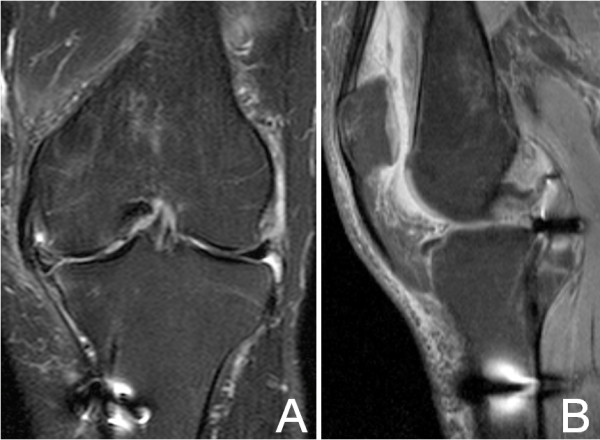
**MRI in T1 sequences 10 months after CMI implantation.** In coronal plane (6**A**) the meniscal complex remains in contact with medial collateral ligament and triangular meniscal shape is observed. In the sagittal plane (6**B**) posterior graft lies completely over the posterior tibial plateau.

Ten months after the placement of the CMI, the patient only complained of occasional discomfort related with periods of increased physical activity. MRI showed reduced synovitis and no osteoarthritis progression neither posterior extrusion of the implant were observed (Figure 6A and 6B).

**Figure 7:**
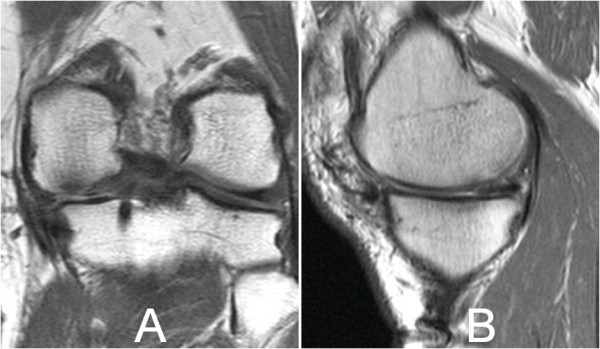
**MRI in T2 at final follow-up.** MRI in coronal (7**A**) and parasagittal (7**B**) planes in T2 sequences showing the correct fitting of the meniscal complex implantation at 24 months. In the coronal plan (7**A**) size and shape are preserved at last follow up. A mild anterior extrusion is seen in the sagittal plane (7**B**).

After two years of the CMI surgery, the patient had no medial compartment pain neither activity daily living limitations and only complained of non specific discomfort with intense activities. *In the Lysholm-Gillquist score 92 points was obtained. The varus deformity was not increased. The MRI revealed the correct fitting and complete integration of implants except moderate extrusion of the anterior horn. No significant degenerative changes were observed. (Figure*7*A and*7*B).*

## Discussion

As described by Bullough [15], most of the collagen fibers in the meniscus are arranged circumferentially to withstand the tension loads. Parallel fibers in the peripheric area can make a very significant difference to strength. The menisci are attached to the joint capsule by the thick convex-shaped peripheral rim, which has a length of 111 mm including the length of the insertional ligaments [16].

Although it is known that the meniscus has some capacity to regenerate after meniscectomy [17], the remaining meniscal structure does not always have enough tissue to support the meniscal implant. In fact, the absence of meniscal wall is a relative contraindication for the collagen meniscus implant, determining poor results in MRI in meniscal transplantation, like the extrusion phenomenon [18], perhaps because it alters the anatomical position of the meniscus or secondary to a capsular laxity, especially in the medial compartment [14,19]. Meniscus extrusion is defined as when it extends beyond the tibial margin. In the non-operated knee, this phenomenon is associated with meniscal injuries and osteoarthritis [16]. Studies in the short and medium term follow-up have reported that meniscal extrusion is correlated with poor results in patients undergoing meniscal transplantation, however it is not well known whether these findings will produce degenerative changes in the long term follow-up [13,18,20].

A surgical technique has been developed for capsular reconstruction in a case with complete absence of the meniscus, consisting in a plication of the capsule with anchors [21]. However, in our opinion, it is not a matter of decreasing the capacity of the joint by plication of the capsule rather than *creating a meniscus rim-like structure that could hold a meniscal implant or transplant.*

*Several surgical techniques can be found to compensate the complete absence of meniscus such as autograft meniscus transplantation in human models*[10,12,22]*with variable results in histologic transformation and mechanical properties.* These studies showed no clinical application except one, where the patellar tendon was used in 20 patients obtaining promising clinical results [12], although these findings were not reconfirmed in other series. The autogenous tendon nature as donor tissue has the obvious benefit of compatibility with a low biological risk [12]. In our opinion, an isolated semitendinosus graft has no biological capacity to become a meniscal fibrocartilage and recreate the anatomy and function of a native meniscus. However, if the tendon graft is complemented with a meniscal implant this complex could generate fibrocartilage tissue similar to the native meniscus. It has been shown that progenitor cells invade the meniscal scaffold from the synovial tissue [23]. Moreover, the CMI has obtained successful clinical and radiological results in the short and long term [5-8], showing that degenerative processes of the joint are not developed after more than 10 years of follow-up [7].

In the surgical technique developed by Johnson [10] the graft is fixed to the stump of the posterior horn of the lateral meniscus and anterior horn through a bone tunnel fixed with a small metal clip. In our technique, the decision was made to drill the bone tunnel towards the posterior horn footprint for the reconstruction of the posterior horn attachment. *This arrangement resists the “hoop stresses” and prevents extrusion of the meniscus with weight bearing. Anterior extrusion could be explained by this fact. Therefore, anterior tibial tunnel is recommended.*

The technique proposed may also be performed in meniscal transplantation without rim, so the meniscal extrusion phenomenon would be minimized or avoided. *In the present case, a collagen meniscus implant was indicated instead of a meniscal allograft transplantation because the meniscal defect was only 40 mm and a normal posterior horn was reconstructed with the present technique.* The meniscus transplantation could have a “biological” advantage over the collagen implant. However, both autografts and allografts are based on meniscal regeneration when used as biological scaffolds as same as the collagen implants [24]. In subsequent studies with MRI, no decrease in size was found and the signal intensity was decreasing over time as indicated by Bugheroni et al. [5]. Other degenerative signs such as the impingement compartment or progression of the chondral damage were not developed over the 2 year follow-up period.

Finally, the tissue engineering applied to the meniscus using meniscal cells in the matrix could be an effective alternative to stimulate the regeneration of fibrocartilage of these scaffolds in order to obtain macroscopic tissue with histological and biomechanical characteristics similar to native meniscus, as demonstrated in the animal model [25-27].

## Conclusion

The semitendinosus tendon autograft seems to be a suitable alternative for enhancing the meniscal wall and posterior horn attachment after a total meniscectomy where a meniscal replacement is not indicated. This technique allows the incorporation of a collagen matrix with good clinical results and satisfactory MRI findings. Nevertheless, a long-term clinical case study must be design in order to provide clinical validity.

## Consent

Written informed consent was obtained from the patient for publication of this Case report and any accompanying images. A copy of the written consent is available for review by the Editor-in-Chief of this journal.

## Abbreviations

CMI: Collagen meniscal implant; MRI: Magnetic resonance image.

## Competing interest

The authors have not received any financial support and have no conflict of interest to report in the preparation of this manuscript.

## Authors’ contributions

JDAM conceived the study and participated in coordination of the manuscript. RCAS collected data and draft the manuscript. MPEM selected the appropiate patient. LAPE developed the surgical technique. All authors read and approved the final manuscript.

## Authors’ information

JDAM. Member of the MTSG (meniscus transplantation study group). Department of Orthopaedic Surgery. Hospital San Rafael. Madrid (Spain).

RCAS. Department of Orthopaedic Surgery, Hospital Asepeyo, Madrid (Spain).

MPEM. Department of Orthopaedic Surgery, Hospital Infanta Leonor. Madrid (Spain).

LAPE. Senior surgeon. Sanatorio Quirúrgico Virgen del Mar, Madrid (Spain).

## Pre-publication history

The pre-publication history for this paper can be accessed here:

http://www.biomedcentral.com/2052-1847/5/4/prepub
